# Failure to prescribe pneumocystis prophylaxis is associated with increased mortality, even in the cART era: results from the Treat Asia HIV observational database

**DOI:** 10.1186/1758-2652-15-1

**Published:** 2012-01-26

**Authors:** Poh-Lian Lim, Jialun Zhou, Rossana A Ditangco, Matthew G Law, Thira Sirisanthana, Nagalingeswaran Kumarasamy, Yi-Ming A Chen, Praphan Phanuphak, Christopher KC Lee, Vonthanak Saphonn, Shinichi Oka, Fujie Zhang, Jun Y Choi, Sanjay Pujari, Adeeba Kamarulzaman, Patrick CK Li, Tuti P Merati, Evy Yunihastuti, Liesl Messerschmidt, Somnuek Sungkanuparph

**Affiliations:** 1 Tan Tock Seng Hospital, Singapore; 2 The Kirby Institute, The University of New South Wales, Sydney, Australia; 3 Research Institute for Tropical Medicine, Manila, Philippines; 4 Research Institute for Health Sciences, Chiang Mai University, Chiang Mai, Thailand; 5 YRG Centre for AIDS Research and Education, Chennai, India; 6 Taipei Veterans General Hospital and AIDS Prevention and Research Centre, National Yang-Ming University, Taipei, Taiwan; 7 HIV-NAT/Thai Red Cross AIDS Research Centre, Bangkok, Thailand; 8 Hospital Sungai Buloh, Kuala Lumpur, Malaysia; 9 National Center for HIV/AIDS, Dermatology & STDs, Phnom Penh, Cambodia; 10 National Center for Global Health and Medicine, Tokyo, Japan; 11 Beijing Ditan Hospital, Beijing, China; 12 Department of Internal Medicine and AIDS Research Institute, Yonsei University College of Medicine, Seoul, Korea; 13 Institute of Infectious Diseases, Pune, India; 14 University of Malaya Medical Centre, Kuala Lumpur, Malaysia; 15 Queen Elizabeth Hospital, Hong Kong, China; 16 School of Medicine Udayana University & Sanglah Hospital, Denpasar, Bali, Indonesia; 17 Working Group on AIDS Faculty of Medicine, University of Indonesia/Ciptomangunkusumo Hospital, Jakarta, Indonesia; 18 TREAT Asia/amfAR (The Foundation for AIDS Research), Bangkok, Thailand; 19 Faculty of Medicine Ramathibodi Hospital, Mahidol University, Bangkok, Thailand

## Abstract

**Background:**

Pneumocystis jiroveci pneumonia (PCP) prophylaxis is recommended for patients with CD4 counts of less than 200 cells/mm^3^. This study examines the proportion of patients in the TREAT Asia HIV Observational Database (TAHOD) receiving PCP prophylaxis, and its effect on PCP and mortality.

**Methods:**

TAHOD patients with prospective follow up had data extracted for prophylaxis using co-trimoxazole, dapsone or pentamidine. The proportion of patients on prophylaxis was calculated for each calendar year since 2003 among patients with CD4 counts of less than 200 cells/mm^3^. The effect of prophylaxis on PCP and survival were assessed using random-effect Poisson regression models.

**Results:**

There were a total of 4050 patients on prospective follow up, and 90% of them were receiving combination antiretroviral therapy. Of those with CD4 counts of less than 200 cells/mm^3^, 58% to 72% in any given year received PCP prophylaxis, predominantly co-trimoxazole. During follow up, 62 patients developed PCP (0.5 per 100 person-years) and 169 died from all causes (1.36/100 person-years). After stratifying by site and adjusting for age, CD4 count, CDC stage and antiretroviral treatment, those without prophylaxis had no higher risk of PCP, but had a significantly higher risk of death (incident rate ratio 10.8, p < 0.001). PCP prophylaxis had greatest absolute benefit in patients with CD4 counts of less than 50 cells/mm^3^, lowering mortality rates from 33.5 to 6.3 per 100 person-years.

**Conclusions:**

Approximately two-thirds of TAHOD patients with CD4 counts of less than 200 cells/mm^3 ^received PCP prophylaxis. Patients without prophylaxis had significantly higher mortality, even in the era of combination ART. Although PCP may be under-diagnosed, these data suggest that prophylaxis is associated with important survival benefits.

## Background

Pneumocystis jiroveci pneumonia (PCP) remains a major cause of morbidity and mortality among HIV-infected persons presenting with advanced infection [1]. Although PCP rates have dropped in the combination antiretroviral therapy (cART) era from 29.9 (1994-1997) to 3.9 (2003-2007) per 1000 person-years in a US cohort [2], PCP mortality still carries a relative mortality hazard of 2.8, even after adjusting for cART, demographics, CD4 cell count and viral load [2].

PCP prophylaxis has been considered the standard of care for patients with CD4 counts of less than 200 cells/mm^3 ^for more than two decades, and current guidelines still support its use for that indication [3,4]. Co-trimoxazole (trimethoprim-sulfamethoxazole, TMP-SMX) remains the first-line agent recommended for Pneumocystis prophylaxis, and has the advantage of being off-patent, inexpensive and widely available. However, in spite of guidelines and the availability of a relatively affordable first-line agent, the use of PCP prophylaxis remains variable in clinical practice in resource-limited settings.

Some studies suggest that after initiation of cART, primary and secondary PCP prophylaxis can be discontinued for patients with CD4 counts of less than 200 cells/mm^3 ^who have achieved virological suppression [5-7]. While these data may support early discontinuation of prophylaxis for a subset of patients with CD4 counts of less than 200 cells/mm^3 ^in developed countries, it is not clear if early discontinuation can be safely practiced in developing countries. There is also relatively little data describing what proportion of patients fall into this subset and the clinical outcomes for not receiving PCP prophylaxis, especially in different geographic settings in the developing world.

Co-trimoxazole has efficacy against a wide range of protozoal and bacterial infections, including toxoplasmosis, isosporosis, malaria, salmonellosis, nocardiosis, and pneumococcal disease. Therefore, in addition to its protective effect against PCP, mortality differences might be observed due to the activity of co-trimoxazole against these other important pathogens among immunocompromised patients, even if some of these are non-AIDS-defining illnesses. This beneficial impact is potentially greater in resource-limited settings where pneumococcal vaccination rates for HIV-infected persons are low, countries with a heavy disease burden of malaria, or countries with higher rates of diarrhoeal illness. Walker and colleagues showed that co-trimoxazole prophylaxis significantly reduced mortality and malaria among HIV-infected adults who were on cART in Africa [8].

This study examines the proportion of HIV-infected patients who were receiving antiretroviral treatment and care in the TREAT Asia HIV Observational Database (TAHOD) with CD4 cell counts of less than 200 cells/mm^3 ^who did not receive PCP prophylaxis, and its effect on PCP and mortality. The purpose of this study was to describe PCP prophylaxis practice in the Asia-Pacific region, and to understand the potential impact of any gaps between guidelines and practice on clinically important outcomes.

## Methods

Established in 2003, TAHOD is a collaborative observational cohort study involving 19 sites in the Asia-Pacific region (see Acknowledgements). Detailed methods have been published previously [9]. Briefly, each site recruited approximately 200 to 300 HIV-infected patients, including patients on cART and those not initiating antiretroviral treatment. Recruitment was based on a consecutive series of patients regularly attending a given site from a particular start-up time. Ethical approval for the study was obtained at participating sites, the data management centre (The Kirby Institute, University of New South Wales, Sydney, Australia), and the coordinating centre (TREAT Asia/amfAR - The Foundation for AIDS Research, Bangkok, Thailand).

The data collected in TAHOD include patient demographics, CD4 and CD8 count, HIV viral load level, prior and new AIDS-defining illness, date and cause of death, prior and current prescribed antiretroviral treatment (ART) and prophylaxis used (with dates of both start and stop). Data on treatment for opportunistic infection were not collected. Data on severe adverse events and routine laboratory testing were also collected, depending on the availability, from each participating sites. AIDS-defining illness, including PCP, was defined according to 1993 Centers for Disease Control and Prevention (CDC) revision of the AIDS case definition [10].

Specifically, a definitive PCP diagnosis is by microscopy (histology), while a presumptive diagnosis is based on: (i) a history of dyspnea on exertion or non-productive cough of recent onset (within the past three months); AND (ii) chest x-ray evidence of diffuse bilateral interstitial infiltrates or evidence by gallium scan of diffuse bilateral pulmonary disease; AND (iii) response to empirical anti-PCP therapy. Data are submitted according to a common protocol. Upon recruitment, all available data prior to entry to TAHOD (considered as retrospective data) are extracted from patient case notes. Prospective data are updated six monthly at each clinic and transferred to the data management centre for aggregation and analyses.

TAHOD patients were included in this analysis if they had prospective clinical visits after enrolment. Prophylaxis data using co-trimoxazole, dapsone or aerosolized pentamidine (AP) were extracted. Prophylaxis data were checked again in each TAHOD site during the study period to make sure the data were most reliable. Dosage on prophylaxis is not collected in TAHOD. Prescriptions of prophylaxis, as well as antiretroviral treatment, are according to the local clinical guidelines. TAHOD sites are encouraged to contact patients who were not seen in the clinics in the previous 12 months.

The baseline date for this analysis was defined as the date the patient was enrolled in TAHOD, and all patients with at least one prospective follow-up period were included. At enrolment, 171 patients had been diagnosed with PCP, and these patients were subsequently excluded in analysis for PCP diagnosis. For analysis on all-cause mortality, all patients were included (including the 171 patients with PCP at enrolment).

The proportion of patients on PCP prophylaxis was calculated for each calendar year from 2003, among active patients who were seen in the database, and among patients who had CD4 counts of less than 200 cells/mm^3 ^in each calendar year and by TAHOD sites. Incidence rates of PCP diagnosis and all-cause mortality were calculated after stratification by current use of PCP prophylaxis and current CD4 cell count.

Random-effect Poisson regression with a forward stepwise approach was used to model incidence rate ratios (IRRs) for time to PCP diagnosis and time to all-cause mortality. CD4 cell count, current CDC disease stage, use of PCP prophylaxis and cART were included as time-updated variables. Patients remained untreated until initiating ART. Other variables considered to be included in the analysis were age and sex. The final multivariate model included all covariates that remained significant at the 0.10 level (two sided). All models were stratified for TAHOD sites. Ninety-five percent confidence intervals (CIs) were calculated using the exact Poisson distribution if there were less than 20 events and a normal approximation if there were 20 events or more. Sensitivity analyses were done excluding sites with extremely low proportion of prophylaxis in patients with CD4 counts of less than 200 cells/mm^3^, which yielded similar predictions (data not shown).

Data management and statistical analyses were performed using SAS for Windows (SAS Institute Inc., Cary, NC, USA) and Stata (StataCorp, STATA 10.1 for Windows, College Station, Texas 77845 USA).

## Results

The analysis included a total of 4050 patients who were enrolled in TAHOD and prospectively followed. The median age at enrolment was 36 years (interquartile range, IQR, 31 to 43), with 64% of patients younger than 40 years. The majority were males (71%). At enrolment, 47% of patients were at CDC stage A, 10% at stage B, and 43% at stage C. There were 171 patients (4% of 4050) who were diagnosed with PCP at enrolment. The median CD4 count at enrolment (the latest within six months) was 301 cells/mm^3 ^(IQR 168 to 442). The annual rate of loss to follow up (defined as not seen in clinic in the previous 12 months) was 6.8 per 100 person-years.

Overall, the median CD4 counts (IQR) was 163 (89-261) cells/mm^3 ^when patients started prophylaxis and the median time (IQR) on prophylaxis was 300 (105-560) days. Of all patients in TAHOD, 90% were on combination antiretroviral treatment, while 39% ever received prophylaxis. Among patients who ever received prophylaxis, 94% received antiretroviral treatment, and 21% of the person-years observed were on prophylaxis after initiation of cART. The prescription of prophylaxis varies across the TAHOD sites: median CD4 counts at start of prophylaxis ranged from 87 to 297 cells/mm^3 ^and median time on prophylaxis ranged from 15 to 472 days.

**Figure 1:**
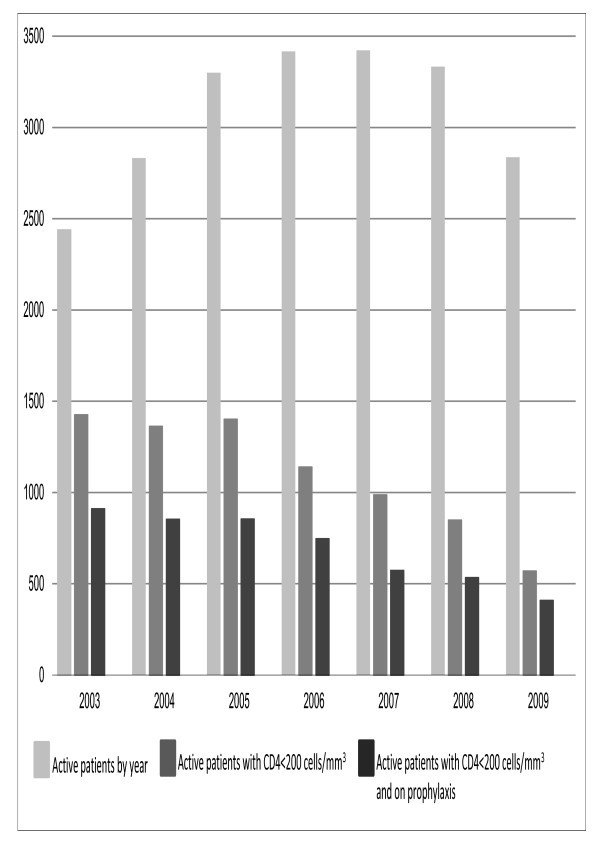
**Number of patients by CD4 count, prophylaxis and calendar year**.

Figure 1 shows the proportions of patients with CD4 counts of less than 200 cells/mm^3 ^and proportions of patients (with CD4 counts of less than 200 cells/mm^3^) who were on PCP prophylaxis by calendar year. In 2003, when TAHOD was established, close to 60% of patients were tested with CD4 counts of less than 200 cells/mm^3^. The proportion decreases steadily between 2003 and 2009, and by 2009, approximately 20% of active patients seen in that year were tested with CD4 counts of less than 200 cells/mm^3^. Among these patients, the proportion of patients on PCP prophylaxis remains stable, approximately 60%, ranging from 58% in 2007 to 72% in 2009. Across TAHOD sites, prescription of prophylaxis varies, ranging from less than 30% to close to 100% in patients who had CD4 counts of less than 200 cells/mm^3^, but results were consistent within sites. Co-trimoxazole was the predominant agent used for PCP prophylaxis (92% of all episodes), compared with dapsone (7%) and AP (2%).

**Figure 2:**
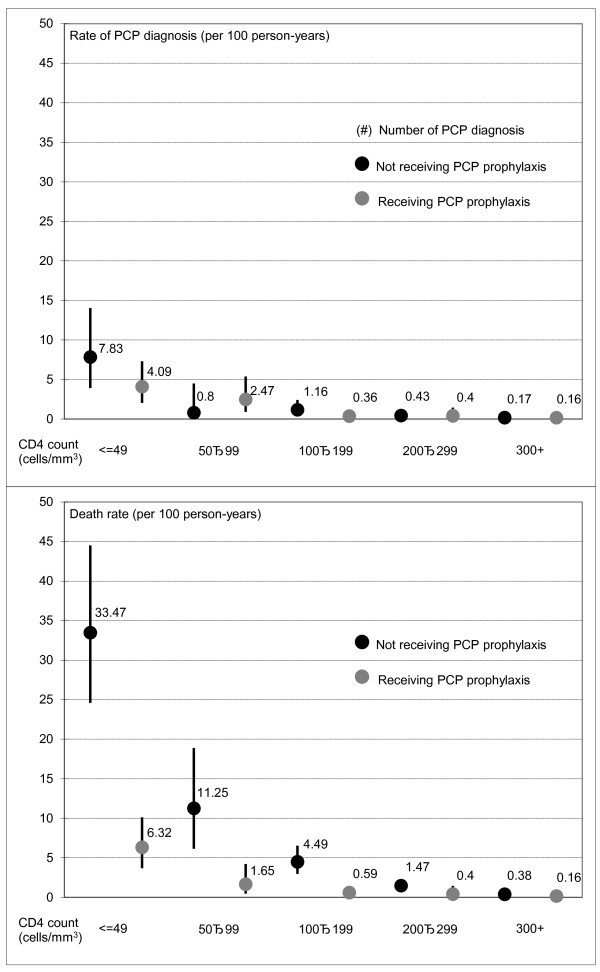
**Rate of PCP diagnosis and death by prophylaxis and CD4 count**.

A total of 62 PCP cases were diagnosed prospectively, a rate of 0.50 per 100 person-years (95% CI 0.39-0.64). Among these PCP cases, 44 were diagnosed as definitive PCP, 14 were presumptive, and four cases were unknown diagnosis type. Current CD4 count was the only predictor of the risk of PCP diagnosis. Table 1 shows the adjusted incident rate ratio (IRR) of PCP prophylaxis in all patients and in patients with CD4 counts of less than 200 cells/mm^3^. In both models, the risks of PCP diagnosis were similar whether or not a patient was receiving PCP prophylaxis (adjusted IRR 0.95, p = 0.901 among all patients; adjusted IRR 1.39, p = 0.560 among patients with CD4 counts of less than 200 cells/mm^3^). As shown in the upper half of Figure 2, the risk of PCP diagnosis, although similar between receiving or not receiving PCP prophylaxis in each CD4 count category, was higher in patients with lower current CD4 counts, particularly those with CD4 counts of less than 50 cells/mm^3^.

**Table 1:** The effect of PCP prophylaxis on PCP diagnosis (adjusted by CD4 count)

	Number of patients*	PCP diagnosis	Person times (year)	Rate (per 100 person-years)	adjusted IRR**	95% CI	p value
Among all patients without PCP at enrolment (n = 3879)							
**Receiving PCP prophylaxis**							
Yes	1567	23	2498.02	0.92	1.00		
No	3439	39	9942.69	0.39	0.95	(0.397, 2.255)	0.901
**CD4 count (cells/mm^3^)**							
< = 49	442	20	336.69	5.94	1.00		
50~99	513	7	366.91	1.91	0.17	(0.053, 0.551)	0.003
100~199	1352	10	1446.18	0.69	0.06	(0.020, 0.170)	< 0.001
200~299	1856	9	2130.43	0.42	0.02	(0.008, 0.073)	< 0.001
300+	3042	14	8088.58	0.17	0.01	(0.003, 0.024)	< 0.001
Not available	120	2	71.91	2.78	0.16	(0.020, 1.249)	0.080
Among patients without PCP at enrolemnt and with CD4 count < 200 cells/μL (n = 1592)							
**Receiving PCP prophylaxis**							
Yes	1109	18	1309.44	1.37	1.00		
No	955	19	840.34	2.26	1.39	(0.463, 4.142)	0.560
**CD4 count (cells/mm^3^)**							
< = 49	442	20	336.69	5.94	1.00		
50~99	513	7	366.91	1.91	0.18	(0.056, 0.556)	0.003
100~199	1352	10	1446.18	0.69	0.05	(0.016, 0.143)	< 0.001

* Patients could contribute to more than one categories.

** Both models were stratified by TAHOD sites.

There were 169 patient deaths during prospective follow up, a rate of 1.36 per 100 person-years (95% CI 1.17-1.58). Table 2 shows the predictors of mortality. Mortality was higher in older patients, patients with lower CD4 counts, patients with more advanced disease stage, and patients not receiving triple or more combination therapy (highly active ART). PCP prophylaxis remained statistically significant in the model after adjustment for these predictors. The risk of mortality in patients not receiving PCP prophylaxis is more than 10 times higher than those receiving prophylaxis (adjusted IRR 10.8, p < 0.001). The lower half of Figure 2 plots mortality by CD4 count and PCP prophylaxis, with mortality decreasing as CD4 count increases. However, the data demonstrate a marked benefit for PCP prophylaxis among patients with CD4 counts of less than 200 cells/mm^3^, with the greatest absolute benefit in patients with CD4 less than 50 cells/mm^3^. In this profoundly immunosuppressed group, PCP prophylaxis was associated with significantly lower mortality, from 33.5 per 100 person-years to 6.3 per 100 person-years.

**Table 2:** Predictors of mortality

	Number of patients*	Death	Person times (year)	Rate (per 100 person-years)	adjusted IRR**	95% CI	p value
**Receiving PCP prophylaxis**							
Yes	1567	29	2498.98	1.16	1.00		
No	3439	140	9942.75	1.41	10.83	(6.258, 18.744)	< 0.001
**Age group**							
Age < 40	2585	80	6650.09	1.20	1.00		
Age 40+	2011	89	5791.64	1.54	1.96	(1.287, 2.997)	0.002
**CD4 count (cells/mm^3^)**							
< = 49	442	57	337.69	16.88	1.00		
50~99	513	18	366.94	4.91	0.20	(0.100, 0.400)	< 0.001
100~199	1352	32	1446.18	2.21	0.08	(0.043, 0.141)	< 0.001
200~299	1856	26	2130.43	1.22	0.03	(0.013, 0.049)	< 0.001
300+	3042	29	8088.58	0.36	0.01	(0.003, 0.012)	< 0.001
Not available	120	7	71.91	9.73	0.13	(0.044, 0.384)	< 0.001
**CDC stage**							
Stage A	1889	42	5266.13	0.80	1.00		
Stage B	474	14	1459.02	0.96	1.56	(0.723, 3.355)	0.257
Stage C	1895	113	5716.58	1.98	2.36	(1.423, 3.903)	0.001
**Antiretroviral treatment**							
No ART	843	30	1331.76	2.25	1.00		
Mono/double	96	4	152.1	2.63	0.99	(0.257, 3.792)	0.984
HAART	3597	135	10957.87	1.23	0.38	(0.219, 0.647)	< 0.001

* Patients could contribute to more than one categories.

** Model was stratified by TAHOD sites.

## Discussion

In this cohort of 4050 HIV-infected patients, a substantial proportion of patients (from 58% in 2003 to 20% in 2009) had advanced immunosuppression with CD4 counts of less than 200 cells/mm^3^, and PCP prophylaxis was indicated for them. However, only two-thirds of these patients received PCP prophylaxis throughout the period. Patients without prophylaxis had a significantly higher mortality, with the effect being most pronounced in those with the lowest CD4 cell counts. However, PCP rates were not significantly higher among those not receiving PCP prophylaxis. It is possible the cohort size was insufficient to demonstrate a significant difference in this clinical endpoint. PCP could also have been under-diagnosed because a definitive diagnosis requires confirmatory laboratory evidence, typically on respiratory samples obtained via bronchoalveoloar lavage. PCP can also be misdiagnosed as pneumonia caused by other etiologies, including tuberculosis.

Nevertheless, these data suggest that PCP prophylaxis, predominantly the use of co-trimoxazole, is associated with important survival benefits. In the pre-cART era, the risks of developing an initial episode of PCP in patients with CD4 counts of less than 200 cells/mm^3 ^by six, 12 and 36 months were 13%, 24% and 39%, respectively [11]. The pooled risk ratio of PCP prophylaxis against PCP events, PCP-related death and all deaths was 0.32, 0.34 and 0.83, respectively, with co-trimoxazole acknowledged to be the most effective agent for PCP prophylaxis (compared with aerosolized pentamidine and dapsone) [12].

Among patients receiving cART, giving prophylaxis until CD4 counts are more than 200 cells/mm^3 ^was estimated to prevent 343 cases of primary PCP per 1000 patients, costing US$5100 per quality-adjusted life-year gained compared with no prophylaxis [13]. A study in Taiwan in patients on cART showed that PCP incidence was 2.81 per 100 person-years among 521 patients who did not initiate or discontinued PCP prophylaxis before achieving CD4 counts over 200 cells/mm^3^, significantly higher than the PCP incidence of 0.45 per 100 person-years among those who continued prophylaxis until CD4 counts were more than 200 cells/mm^3 ^(adjusted risk ratio 5.32) [7].

In our study, this mortality benefit was observed even among patients with CD4 counts over 200 cells/mm^3^. Unlike the DART African cohort, TAHOD patients reside in the Asia-Pacific region, with almost all TAHOD sites located in urban centres, which are malaria-free for the most part. The mortality benefit of co-trimoxazole prophylaxis therefore cannot be readily explained by its effect on malaria. It is possible that co-trimoxazole reduces mortality by its protective efficacy against an array of other pathogens for which HIV-infected persons with the lowest CD4 cell counts are at higher risk, such as cerebral toxoplasmosis, salmonellosis or pneumococcal disease. A higher incidence rate of bacterial infections (14.48 per 100 person-years) was observed among the Taiwan patients on cART with CD4 counts of less than 200 cells/mm^3 ^who were not on PCP prophylaxis compared with an incidence rate of 5.56 per 100 person-years for those taking prophylaxis [14]. The role of vaccination and its poor uptake in resource-limited settings is often overlooked, but if pneumococcal vaccination rates are low for most patients who present with advanced HIV infection, this may account for some of the protective effect of co-trimoxazole prophylaxis on mortality.

It is possible that PCP prophylaxis prescribing behaviour might be a marker of better quality of care, or provider compliance with treatment guidelines. Another possible explanation for the mortality difference observed between those who received PCP prophylaxis compared with those who did not could be that patients who were unable to tolerate co-trimoxazole or dapsone prophylaxis due to rash or other toxicities and were less able to afford the more expensive third-line agent, aerosolized pentamidine, were more likely to go without PCP prophylaxis. Socio-economic factors might therefore act as potential confounders affecting the outcomes studied.

Among other explanations for why almost one-third of patients with appropriate indications did not receive PCP prophylaxis was the issue of high pill burden, especially in those with the lowest CD4 cell counts. This was cited by some clinicians when queried about low rates of prophylaxis from their sites. It is possible therefore that the effect of concomitant opportunistic infections (for which high pill burdens are a marker) could account for the difference in mortality between those with and without PCP prophylaxis.

Other studies have examined the risk of discontinuing PCP prophylaxis in patients with CD4 counts of less than 200 cell/mm^3 ^who had achieved virological suppression. However, the studies are not directly comparable; the COHERE study, conducted in European cohorts, had as its primary endpoint incidence of primary PCP rather than mortality, and viral load measurements were generally accessible [14]. In our analysis, HIV viral loads did not appear to independently predict mortality, but this should be interpreted with caution because HIV viral load measurements were missing in a large proportion of patients and are not routinely accessible because of resource limitations. However, the different findings between European, African and Asian cohorts may highlight important epidemiological and clinical differences between regions.

Our finding that almost one-third of this cohort with the appropriate CD4 indication did not receive PCP prophylaxis was somewhat unexpected because the TAHOD sites comprise major urban academic centres for HIV care and often represent the leading national HIV treatment centres in their respective countries. Some clinicians cited anticipation that patients' CD4 cell counts would rise quickly with cART treatment, and wishing to avoid causing rash or other toxicity in patients taking multiple medications. The precise reasons for not prescribing prophylaxis in individual HIV-infected patients were hard to examine, and retrospective collection of this information would risk introducing recall bias. More specific study plans should be developed, aiming at prescription practice, compliance with and tolerability of prophylaxis against PCP and other opportunistic infections.

Several limitations should be considered in interpreting the results in this study. TAHOD subjects were recruited from patients who were considered by site investigators to be good candidates for long-term follow-up. The patients in this cohort and their treatment, therefore, may not be truly representative of all HIV-infected patients in the Asia-Pacific region. In addition, data on prophylaxis adherence were not collected in TAHOD. Allowing for these limitations and extrapolating from this data, one might speculate that PCP prophylaxis rates for other HIV treatment centres could be even lower. There were a small proportion of patients with missing or unavailable CD4 count information, which would, to some extent, increase the uncertainty of our final models.

Lastly, due to the observational nature of TAHOD, there are possible unmeasured confounders associated with prophylaxis. As a consequence, our findings could overestimate the benefit of prophylaxis, even after adjustments and stratification. We therefore recommend that the interpretation of our data be made cautiously.

## Conclusions

Patients without prophylaxis had significantly higher mortality, even in the era of combination ART, suggesting that prophylaxis is associated with important survival benefits. Our data highlight an important gap between guidelines and actual practice that represents a missed opportunity for prevention. Implementing PCP prophylaxis according to accepted guidelines could have a substantial impact on mortality for HIV-infected populations in Asia, as well as Africa. These findings also suggest that we may wish to proceed with caution when calling for discontinuation of PCP prophylaxis for patients who have CD4 cell counts of less than 200 cells/mm^3^, even with virological suppression. When developing protocols and guidelines for prophylaxis against opportunistic infections, it may be prudent to be aware of potential differences between different geographic regions and access to resources.

## Competing interests

The authors declare that they have no competing interests.

## Authors' contributions

PLL undertook study conception, data interpretation and drafting of the manuscript. JZ was responsible for study design and data analysis. RD was responsible for data acquisition and interpretation and critical revision of the manuscript. MGL undertook study design, data interpretation and critical revision of the manuscript. TS, NK, YMAC, PP, CKCL, VS, SO, FZ, JYC, SP, AK, PCKL, TPM, EY, LM and SS were responsible for data acquisition and interpretation and critical revision of the manuscript. All authors have read and approved the final version of this manuscript.

## References

[B1] Buchasz K Baker RK Palella FJ Chmiel JS Lichtenstein KA Novak RM Wood KC Brooks JT the HOPS investigators AIDS-defining opportunistic illnesses in US patients, 1994-2007: a cohort study AIDS 2010 15 10 1549 1559 10.1097/QAD.0b013e32833a3967 20502317

[B2] Antiretroviral Therapy Cohort Collaboration (ART-CC) Variable impact on mortality of AIDS-defining events diagnosed during combination antiretroviral therapy: Not all AIDS-defining conditions are created equal Clin Infect Dis 2009 15 1138 1151 10.1086/597468 PMC3032444 19275498

[B3] Guidelines for prevention and treatment of opportunistic infections in HIV-infected adults and adolescents. Recommendations from CDC, the National Institutes of Health (NIH), the Centers for Disease Control and Prevention (CDC), and the HIV Medicine Association of the Infectious Disease Society of America (HIVMA/IDSA) Morbidity and Mortality Weekly Report (MMWR) 2009 15 RR4 19357635

[B4] AIDS Study Group (GESIDA) 2008 prevention of opportunistic infections in HIV-infected adolescents and adults guidelines. Recommendations of GESIDA/National AIDS Plan AIDS Study Group Enferm Infecc Microbiol Clin 2008 15 437 464 10.1157/13125642 18842240

[B5] Weverling GJ Mocroft A Ledergerber B Kirk O Gonzalez-Lahoz J Monforte AD Lundgren JD Reiss for the EuroSIDA Study Group Discontinuation of Pneumocystis carinii pneumonia prophylaxis after start of highly active antiretroviral therapy in HIV-1 infection Lancet 1999 15 1293 8 10.1016/S0140-6736(99)03287-0 10218526

[B6] D'Egidio GE Kravcik S Cooper CL Cameron DW Fergusson DA Angel JB Pneumocystis jiroveci pneumonia prophylaxis is not required with a CD4+ T-cell count less than 200 cells/microl when viral replication is suppressed AIDS 2007 15 1711 1715 17690568 10.1097/QAD.0b013e32826fb6fc

[B7] Cheng CY Hung CC Chen MY Hsieh SM Sheng WH Sun HY Lo YC Liu WC Risk of pneumocystosis after early discontinuation of prophylaxis among HIV-infected patients receiving highly active antiretroviral therapy BMC Infect Dis 2010 15 126 133 10.1186/1471-2334-10-126 20492660 PMC2885390

[B8] Walker AS Ford D Gilles CF Munderi P Ssali F Reid A Katabira E Grosskurth H Mugyenyi P Hakim J Darbyshire JH Gibb DM Babiker AG Daily cotrimoxazole prophylaxis in severely immunosuppressed HIV-infected adults in Africa started on combination antiretroviral therapy: an observational analysis of the DART cohort Lancet 2010 15 1278 1286 10.1016/S0140-6736(10)60057-8 20347483 PMC2858802

[B9] Zhou J Kumarasamy N Predicting short term disease progression among HIV-infected patients in Asia and the Pacific region: preliminary results from the TREAT Asia HIV Observational Database (TAHOD) HIV Med 2005 15 216 223 10.1111/j.1468-1293.2005.00292.x 15876289 PMC10480320

[B10] Centers for Disease Control and Prevention 1993 revised classification system for HIV infection and expanded surveillance case definition for AIDS among adolescents and adults Morbidity and Mortality Weekly Report (MMWR) 1992 15 RR-17 1 19 1361652

[B11] Polk BF Fox R Brookmeyer R Kanchanaraksa S Kaslow R Visscher B Rinaldo C Phair J Predictors of acquired immunodeficiency syndrome developing in a cohort of seropositive homosexual men N Engl J Med 1987 15 61 66 10.1056/NEJM198701083160201 3024007

[B12] Ioannidis JP Cappelleri JC Skolnik PR Lau J Sacks HS A meta-analysis of the relative efficacy and toxicity of *Pneumocystis carinii *prophylactic regimens Arch Intern Med 1996 15 177 188 10.1001/archinte.1996.00440020081010 8546551

[B13] Goldie SJ Kaplan JE Losina E Weinstein MC Paltiel AD Seage GR Craven DE Kimmel AD Zhang H Cohen CJ Freedberg KA Prophylaxis for Human Immunodeficiency Virus-related *Pneumocystis carinii *pneumonia Arch Intern Med 2002 15 921 928 10.1001/archinte.162.8.921 11966344

[B14] Opportunistic Infections Project Team of the Collaboration of Observational HIV Epidemiological Research in Europe (COHERE) Mocroft A Reiss P Kirk O Mussini C Girardi E Morlat P Stephan C De Wit S Doerholt K Ghosn J Bucher HC Lundgren JD Chene G Miro JM Furrer H Is it safe to discontinue primary *Pneumocystis jiroveci *pneumonia prophylaxis in patients with virologically suppressed HIV infection and a CD4 cell count < 200 cells/μL? Clin Infect Dis 2010 15 5 611 619 20645862 10.1086/655761

